# Can hypothyroidism be a protective factor for hepatocellular carcinoma in cirrhosis?

**DOI:** 10.1097/MD.0000000000019492

**Published:** 2020-03-13

**Authors:** Tolga Sahin, Alihan Oral, Fatih Turker, Erdem Kocak

**Affiliations:** a Department of Gastroenterology; b Department of Internal Medicine, Faculty of Medicine, Demiroğlu Bilim University, Istanbul, Turkey.

**Keywords:** cirrhosis, hepatocellular carcinoma, hypothyroidism, thyroid hormones

## Abstract

Despite many studies, the molecular mechanisms of hepatocellular carcinoma (HCC) development remain unclear. Thyroid hormone (TH) levels may vary in many chronic diseases including cirrhosis. The aim of this study was to evaluate TH status in patients with cirrhosis and HCC and to investigate the relationship between THs and HCC development.

Five hundred seventy-seven patients with cirrhosis who applied to Demiroğlu Bilim University, Faculty of Medicine, Gastroenterology Department between 2004 and 2019 were included the study. Three hundred sixty-seven patients who applied to Internal Medicine Unit for general health check-up were included in the study as healthy control group. Demographic, laboratory, and imaging findings of study groups were retrospectively reviewed and recorded from hospital information system.

In the cirrhosis group, 252 patients had HCC (43.67%), and 325 patients had non-HCC cirrhosis (56.33%). Free thyroxine (FT4) levels were higher in the control group than in the cirrhotic group but there was no significant difference (*P* = .501). Thyroid-stimulating hormone (TSH) and FT4 levels were similar between groups, while free triiodothyronine (FT3) levels were significantly different between HCC group, non-HCC cirrhosis group, and control group (*P* = .299 for TSH, *P* = .263 for FT4, *P* < .001 for FT3). FT3 levels were significantly higher in HCC group than non-HCC cirrhosis group, but significantly lower than control group (*P* < .05).

Our study confirmed the presence of hypothyroidism in cirrhosis patients and clearly demonstrated a strong relationship between FT3 levels and HCC development.

## 1 Introduction

Hepatocellular carcinoma (HCC) is the second most common cause of cancer-related deaths worldwide, with an estimated 800,000 new cases every year.^[1]^ HCC usually develops in patients with cirrhosis and has many risk factors, such as chronic viral hepatitis B and C, chronic alcohol consumption, diabetes mellitus, obesity, nonalcoholic steatohepatitis (NASH), and aflatoxin.^[2]^ Despite significant improvements in treatment methods, the 5-year survival rate for HCC is less than 12%.^[3]^

Thyroid hormones (THs) are synthesized by the thyroid gland through a multistage mechanism, and the liver has a critical role in TH homeostasis. THs are widely known regulators of growth, metabolism, and development. THs have mitogenic effects on hepatic metabolism by affecting gene expression levels and cell cycle progression. These effects reach beyond genomic pathways by initiating rapid and nongenomic signals. It was found that THs strongly affect liver regeneration by altering the expression and activity of proteins involved in cell cycle control, thereby contributing to liver homeostasis.^[4,5]^ In addition, TH binding proteins are synthesized in the liver, which is the main organ responsible for the conversion of thyroxine (T4) to triiodothyronine (T3) by deiodinase enzymes. Nearly 80% of daily circulating T3 is produced by deiodinase enzymes from T4 in the liver. There are 3 types of deiodinase enzymes in the liver. Deiodinase 1 (DIO1) and deiodinase 2 (DIO2) have major roles in the conversion of T4 to T3 in the liver. Type III deiodinase decreases intracellular TH activity by converting T4 and T3 to inactive metabolites reverse T3 (rT3) and 3,3′-diiodothyronine, respectively. Some TH conversion occurs in the skeletal muscle, kidney, and other organs through the same enzymes.^[6]^

Changes in TH metabolism in chronic liver disease usually manifest as nonthyroidal illness syndrome (NTIS) characterized by low total T3, low free T3 (FT3), increased rT3, normal/low total T4, and normal/high free T4 (FT4) levels. Thyroid function in cirrhosis is also controversial.^[7]^ Although some studies have found an inverse relationship between serum T3 concentrations and the severity of liver dysfunction, many others have not found clinical findings of hypothyroidism in patients with cirrhosis.^[8,9]^

The relationship between thyroid function and HCC development is complicated. Despite strong evidence that T3 stimulates mitosis in hepatocytes,^[10]^ similar findings have not been achieved in HCC cells. Various studies have shown an association between HCC and hypothyroidism,^[11–13]^ but the effect of THs on the development of HCC is unclear. This study aimed to evaluate thyroid function in patients with cirrhosis and to investigate the relationship between TH activity and HCC development.

## 2 Materials and methods

Between January 2004 and February 2019, 577 cirrhosis patients admitted to the gastroenterology outpatient clinic of Demiroğlu Bilim University as liver transplant recipient candidates were included in the study. In addition, 367 patients who presented to the internal medicine department for a general health examination were included in the study as the control group. The control group consisted of individuals older than 18 years with no systemic disease. Ethical committee approval was provided by the Demiroğlu Bilim University Ethical Committee (approval number: 44140529/2019-9541). Demographic (age, sex, height, weight, body mass index [BMI]), laboratory (biochemical parameters), and imaging (ultrasonography, computed tomography, magnetic resonance imaging) findings, and histopathological examination results of the patients stored in the hospital information system were retrospectively reviewed. The chemiluminescence microparticle immunoassay method was used to quantitatively determine serum thyroid-stimulating hormone (TSH), FT3, and FT4 levels of all patients and the control group. HCC was diagnosed in all patients using radiological imaging and histological examinations of hepatectomy materials. Individuals with a history of hypothyroidism or hyperthyroidism, those using active levothyroxine, those younger than 18 years, or those whose laboratory or radiological data were not available were excluded from the study.

### 2.1 Statistical analysis

Numerical data are provided as mean and standard deviation. Data with normal distributions were calculated using the Student *t* test, and data without normal distributions were calculated using the Mann–Whitney *U* test. Categorical data were calculated using the Chi-squared test. The Kruskal–Wallis test and logistic regression analysis were performed. Statistical analyses were performed using SPSS (Chicago, IL) 21.00, and a 95% confidence interval was accepted. *P* < .05 was considered statistically significant.

## 3 Results

### 3.1 Characteristics and laboratory findings of the study population

There were 577 patients in the cirrhosis group and 367 patients in the control group. In the cirrhosis group, 252 patients had both cirrhosis and HCC (43.67%) and 325 patients had cirrhosis without HCC (56.33%). The cirrhosis group consisted of 48.3% female patients and 51.7% male patients. In the control group, 40.3% were female and 59.7% were male. Almost all participants had the same ethnicity. Age, BMI, aspartate aminotransferase, alanine aminotransferase (ALT), gamma-glutamyl transferase (GGT), alkaline phosphatase (ALP), international normalized ratio (INR), and total bilirubin levels were significantly higher in the cirrhosis group than in the control group.

TSH and FT3 levels were significantly lower in the cirrhosis group (*P* = .031 for TSH and *P* < .001 for FT3). FT4 levels were higher in the control group than in the cirrhosis group, but they were not significantly different (*P* = .501). Serum albumin levels were also lower in the cirrhosis group (*P* < .001). Table 1 summarizes the demographic and laboratory findings of the study population.

**Table 1:**
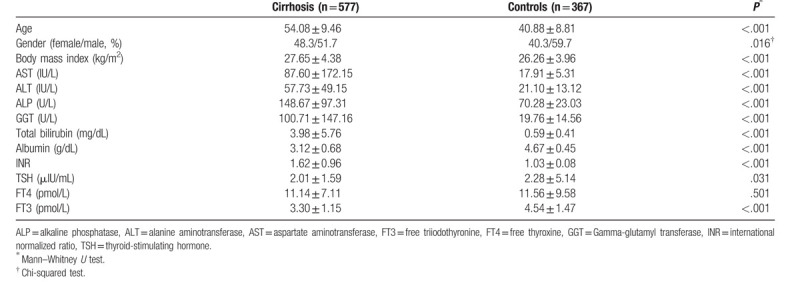
Clinical, laboratory, and demographic data of cirrhosis patients (hepatocellular carcinoma and nonhepatocellular carcinoma) compared to controls.

### 3.2 Comparison of cirrhosis group patients

Cirrhosis patients were divided into 2 groups according to the presence of HCC: 252 patients had HCC and cirrhosis (HCC group) and 325 patients had cirrhosis but not HCC (non-HCC group). Age and male sex ratios were significantly higher in the HCC group (*P* < .001 and *P* = .013, respectively). The alpha fetoprotein (AFP), albumin, and GGT levels were higher in the HCC group; however, total bilirubin, INR, model for end-stage liver disease (MELD) scores, and Child–Pugh scores were higher in the non-HCC group (*P* < .001). When patients were evaluated to determine the TH status, TSH and FT4 levels were higher in the non-HCC group, with no significant differences (*P* = .3 and *P* = .086, respectively), but FT3 levels were significantly higher in the HCC group (*P* < .001) (Table 2).

**Table 2:**
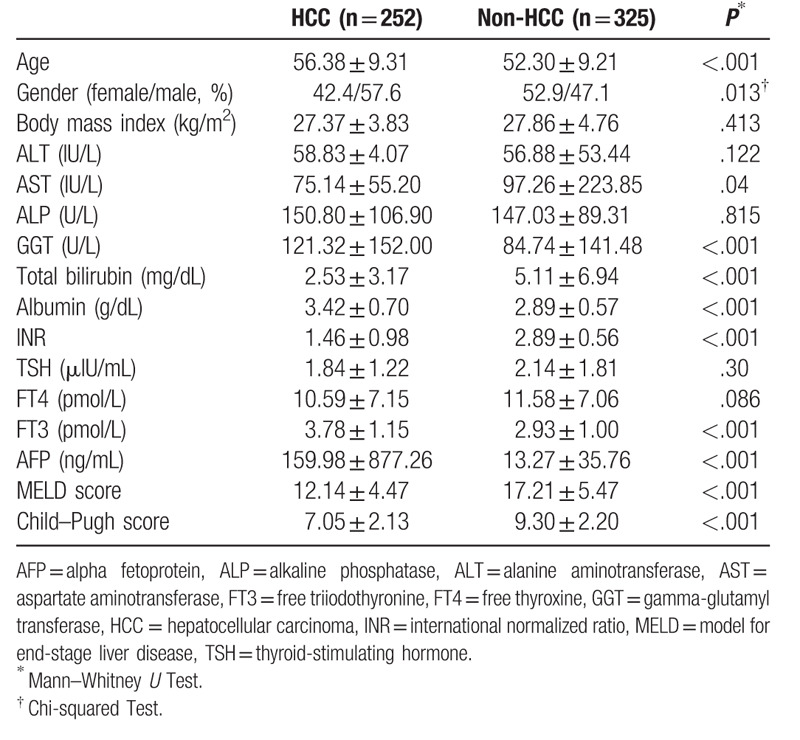
Clinical, laboratory, and demographic data of hepatocellular carcinoma patients compared to nonhepatocellular carcinoma patients with cirrhosis.

### 3.3 Comparison of TH levels among study groups

When study patients were compared in terms of TH levels, TSH and FT4 levels were similar but FT3 levels were significantly different among the HCC group, non-HCC group, and control group (*P* = .299 for TSH; *P* = .263 for FT4; *P* < .001 for FT3). FT3 levels were significantly higher in the HCC group than in the non-HCC group; however, they were significantly lower than those in the control group (*P* < .05). In addition, FT3 levels were significantly lower in the non-HCC group than in the HCC group and control group (*P* < .05) (Table 3).

**Table 3:**
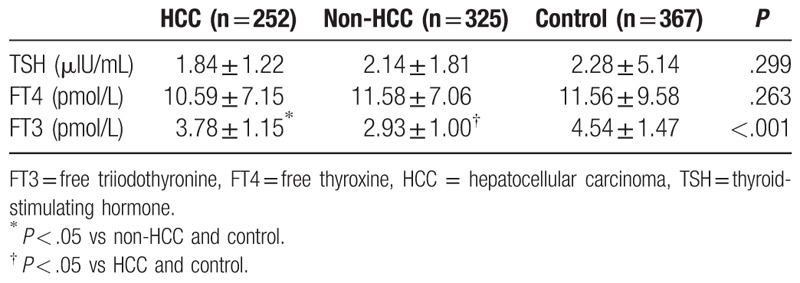
The comparison of thyroid hormones between hepatocellular carcinoma, nonhepatocellular carcinoma, and control group.

### 3.4 Relationship between THs, tumor characteristics, and HCC stage

Patients with cirrhosis and HCC were evaluated to determine the maximum tumor diameter, total tumor diameter, and number of tumors. TSH, FT3, and FT4 levels were not correlated with tumor characteristics (*P* > .05) (Table 4). When HCC patients were divided into low-grade and high-grade HCC groups and examined to determine TH levels, there was no significant relationship among tumor grade, THs, and TSH (*P* = .473 for TSH; *P* = .542 for FT4; *P* = .351 for FT3) (Table 5).

**Table 4:**
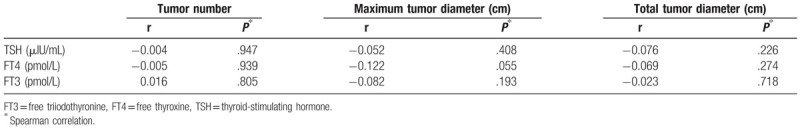
Relationship between thyroid hormones and tumor characteristics.

**Table 5:**
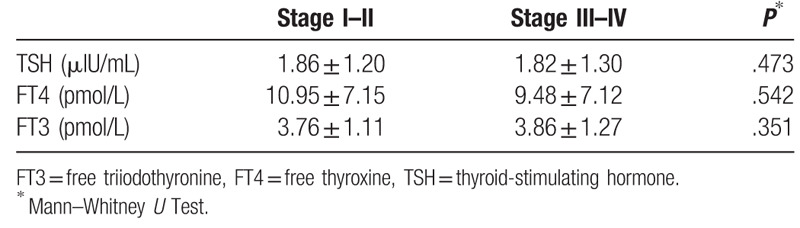
Relationship between thyroid hormones and stage of hepatocellular carcinoma.

### 3.5 Logistic regression analysis

HCC was independently associated with many variables in the logistic regression analysis, including older age, male sex, BMI, ALT, ALP, MELD score, Child–Pugh score, albumin, AFP, and FT3 (Table 6).

**Table 6:**
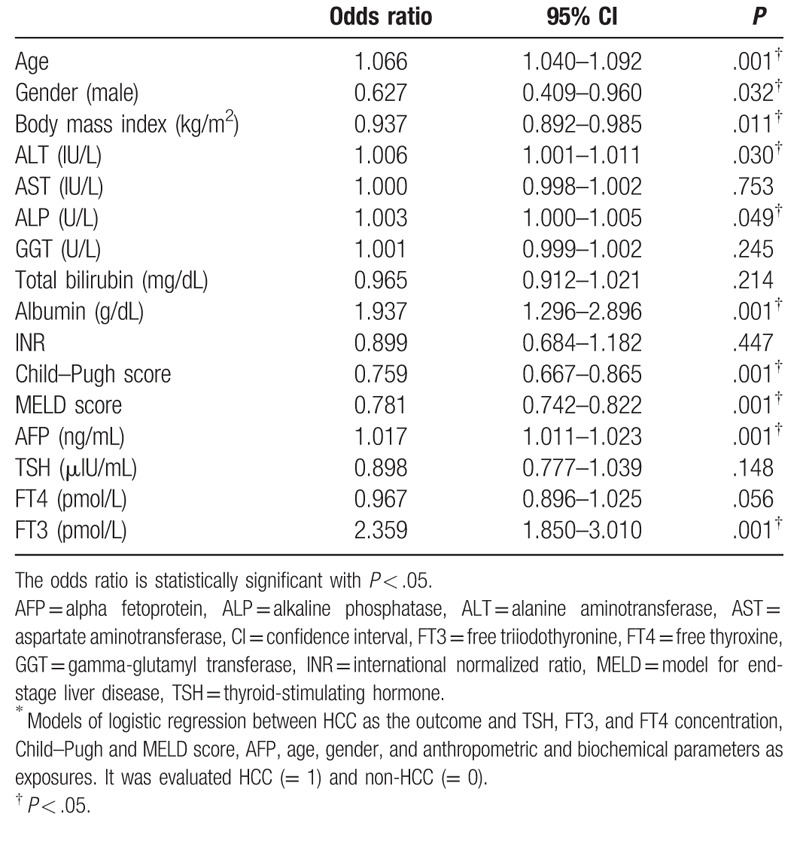
Independent association of variables with hepatocellular carcinoma (logistic regression analysis)^∗^.

## 4 Discussion

Although the relationship among hypothyroidism, cirrhosis, and HCC development has been emphasized in the literature, the issue is still controversial due to the different results obtained by different studies.^[8,9,11–13]^ Our study demonstrated the presence of hypothyroidism in patients with cirrhosis and HCC. Interestingly, our study revealed that hypothyroidism levels were significantly different among patients with cirrhosis and HCC. The lowest FT3 levels were found in the non-HCC group in our study. FT3 levels were lower in the HCC group than in the control group, but they were significantly higher than those of the non-HCC group. Our study showed that hypothyroidism levels are significantly different among patients with cirrhosis and HCC. There are many molecular mechanisms involved in the development of hypothyroidism in cirrhosis. Some studies have suggested that NTIS and low TH levels are an adaptation mechanism used to reduce the basal metabolic rate and caloric requirements of hepatocytes to maintain liver function and total body protein stores.^[7]^ Selenium is an antioxidant element and a critical cofactor in the deiodinase enzyme family in the liver. Selenium levels decreased during chronic liver disease.^[14]^ Decreased selenium levels caused a decrease in DIO1 and DIO2 activity in the liver tissue, which indicated a decrease in the conversion from T4 to T3 in the liver. Decreased activity of deiodinases is the most common cause of NTIS and lower T3 levels in cirrhosis. The liver is the main organ in which many plasma proteins, including thyroxine-binding protein (TBP), are synthesized. Decreased TBP and T4/T3 uptake in chronic liver disease may be another cause of hypothyroidism in cirrhosis. Increased intestinal permeability induced bacterial translocation, leading to opportunistic infections such as spontaneous bacterial peritonitis and sepsis in advanced cirrhosis. Infections and sepsis are the other causes of NTIS and hypothyroidism in cirrhosis.^[15–17]^ This study confirmed decreased TH levels in cirrhosis.

Hypothyroidism is associated with many diseases that may be risk factors for the development of HCC, such as nonalcoholic fatty liver disease,^[18]^ NASH,^[19]^ obesity,^[20]^ diabetes mellitus,^[21,22]^ and chronic hepatitis C infection.^[23,24]^ In a recent study, a long-term history of hypothyroidism has been found to be an independent risk factor for the development of HCC in women.^[11]^ However, the relationship between hypothyroidism and HCC development in men is still unclear. Lower FT3 levels and higher BMI values were found in the HCC group compared to the healthy control group, which was compatible with the results found in the literature.

Hepatocyte apoptosis induced by death receptor ligands (DRLs) such as tumor necrosis factor-α or Fas is known to have an important role in various liver diseases, including alcoholic hepatitis, viral and inflammatory hepatitis, acute liver failure, and ischemia/reperfusion injury.^[25,26]^ Recent studies have demonstrated that T3 has an anti-apoptotic effect by affecting different molecular pathways on hepatocytes. The current literature data have shown that the TH status affects susceptibility to apoptosis and that T3 has a significant role in the survival of hepatocytes because of its strong anti-apoptotic effects after the onset of apoptosis triggered by DRL. Inhibition of Na^+^–H^+^ exchangers (NHEs) in hepatocyte membranes increases intracellular acidity and decreases pH in Fas-induced apoptosis. Acidic pH leads to apoptosis by stimulating endonuclease activity and DNA fragmentation in hepatocytes. T3 removes this inhibition by affecting the NHEs and contributes to the recovery of normal pH in hepatocytes, thus maintaining this optimal pH. T3 can also show anti-apoptotic effects using nongenomic rapid pathways. T3 increases the level of intracellular Cyclic adenosine monophosphate (cAMP) by activating enzymes such as protein kinase A (PKA), protein kinase C, extracellular signal-regulated kinase, and mitogen-activated protein kinase through nongenomic mechanisms. Activation of these enzymes and increases in intracellular cAMP have critical roles in alkalinization of intracellular pH by reactivating NHEs.^[27]^ Additionally, it has been shown that PKA activation and increased cAMP mediate TH effects such as increased growth hormone receptor signaling in cancer cells.^[28,29]^ T3 may increase sensitivity to growth hormones by inducing PKA activation and cAMP increases in hepatocytes, thereby indirectly contributing to the development and progression of HCC.

THs also have serious effects on mitochondrial biogenesis and activity.^[30,31]^ Because the liver is one of the main target organs for THs, it is inevitable that hypothyroidism in cirrhosis has effects on hepatic mitochondrial metabolism and functional integrity. Previous studies have shown that hypothyroidism reduces mitochondrial oxygen consumption and adenosine triphosphate synthesis,^[32]^ and that external T3 injections increase oxygen consumption and metabolic rates.^[33]^ It is known that hypothyroidism in cirrhosis affects mitochondrial oxidative phosphorylation and disrupts the function of many antioxidant enzymes, such as superoxide dismutase and glutathione.^[34]^ Changes in protective antioxidant mechanisms lead to excessive accumulation of free oxygen radicals in hepatocytes. This accumulation may have a critical role in the development of HCC in cirrhosis by causing oxygen free radical-associated DNA damage in hepatocytes.

In some studies, controlled hypothyroidism has been suggested to be a protective mechanism against more liver damage in patients with cirrhosis.^[23,24]^ T3 was found to stimulate hepatocyte proliferation and DNA synthesis in animal models. Preclinical studies have shown that T3-induced cyclin D1 expression increases phosphorylation of Retinoblastoma protein and increases the expression of transcription factor E2F-mediated proliferation on hepatocytes.^[35]^ This finding proves that T3 exerts proliferative effects on liver cells by regulating cell cycle proteins. T3 has potent mitotic activity in hepatocytes, and decreasing FT3 levels may be a protective mechanism against uncontrolled proliferation of hepatocytes.^[36]^ Our study quantitatively showed that T3 activity is significantly increased in HCC compared to that in cirrhosis. Increased FT3 and TH receptor activity may be the main molecular mechanism leading to uncontrolled hepatocyte proliferation and DNA damage in patients with cirrhosis. This damage and uncontrolled proliferation may also trigger the development of HCC.

Wang et al^[37]^ found a strong relationship between TH activity and HCC development and progression. In this study, THs stimulated the regeneration of HCC cells and also increased the percentage of CD90 and HCC cells, thus leading to drug resistance in HCC. They found that thyroid receptor alpha (TRa) expression in patients with metastatic HCC was higher in both HCC and HCC-associated malignant portal vein thrombosis tissues than in normal liver tissues. In the same study, inhibition of TRa was found to reduce self-regeneration and growth of HCC cells.^[37]^ In addition, many other preclinical studies have demonstrated the effects of THs and receptors on the development and progression of HCC through different genetic and biochemical mechanisms.^[38–41]^ This study clearly demonstrated the association of THs, especially T3, with HCC development in cirrhosis. Although TSH and FT4 levels were associated with the prognosis for HCC in some other studies,^[42]^ TSH and FT4 levels did not differ significantly between groups in our study. In addition, FT3 showed a significant correlation with cirrhosis and HCC in our study.

In our study, free T3 levels were significantly higher in patients with cirrhosis who developed HCC than in patients with cirrhosis without HCC. The data obtained in our study and that of other preclinical studies indicate that it is clear that T3 has a critical role in the development of HCC in patients with cirrhosis. Our study is the first clinical study comparing tumor size, tumor grade, and status of THs in patients with HCC. Although the results of our study were consistent with those of many studies, TH levels were not correlated with tumor grade or size in our study. This suggests that different hormonal and molecular mechanisms other than THs may be simultaneously effective in the development and progression of HCC.

Our study had some limitations. The main disadvantages were that it had a retrospective design and was a single-center study that lacked some data, such as serum TBP, total T3, and total T4 levels.

## 5 Conclusion

The presence of hypothyroidism in cirrhosis and HCC patients is a previously highlighted finding in the literature. Our study revealed a different finding in cirrhosis and HCC patients. Our study confirmed the presence of hypothyroidism in patients with HCC and cirrhosis, but found a significant difference in TH activity between the 2 patient groups. This finding showed that increased TH activity may have an effect on HCC development in cirrhosis patients. New and comprehensive studies may fully reveal the molecular mechanisms of HCC in the future and enable curative treatment methods to be developed.

## Author contributions

**Conceptualization:** Tolga Sahin.

**Data curation:** Tolga Sahin, Alihan Oral, Fatih Turker.

**Formal analysis:** Alihan Oral.

**Investigation:** Tolga Sahin, Alihan Oral, Fatih Turker, Erdem Kocak.

**Methodology:** Tolga Sahin, Alihan Oral.

**Writing – original draft:** Tolga Sahin.

**Writing – review and editing:** Tolga Sahin.

Alihan Oral orcid: 0000-0003-1160-9340.

Fatih Turker orcid: 0000-0002-8281-0319.

Erdem Kocak orcid:0000-0001-6675-8963.

Tolga Şahin orcid: 0000-0003-1569-4941.
